# “Efficiency Dentistry” and the “Zero System”

**Published:** 1918-01

**Authors:** Alfred A. Crocker


### "EFFICIENCY DENTISTRY" AND THE "ZERO SYSTEM"

BY ALFRED A. CROCKER

The attention to the teeth is not a vogue of society nor an effort per se to maintain good appearances. While dentistry has a feature of esthetics, it's larger, and it's main demand is at present one of necessity. There has been evolved through the fifty odd years of the development of the present practice of the dental profession, the system now termed "Efficiency Dentistry." A patient can, by periodical visits to the dentist, minimize the decay of the teeth approaching the zero point. The dentist at each periodical visit of the patient, examines the teeth to discover ill health or any unsoundness, and if a cavity is found a filling is made which is the only known way of stopping dental caries. Such a discovery is of course made long before the decay would be advanced enough to cause the patient to notice it because of any pain. Sometimes patients complain of lassitude and lack of energy, but of course, do not attribute it to the slight sensitiveness of a small cavity in a tooth, for at this stage the cavity is often in between the teeth and not observable except to a dentist, and it may not be possible to locate it with the tongue. The average patient does not complain until a tooth aches and of course by that time the decay has reached the sensitive nerve pulp. During the entire progress of a decay in a tooth from the time of the first softening of the enamel until the cavity has enlarged and deepened and it starts to ache, there is an increasing condition of irritability developed until the pain in the tooth announces to the patient the true cause. During this process the patient, if not in touch with a dentist, often resorts to nerve sedatives which, of course, do not rectify the cause in the least and only complicates the condition. By following the *"Zero System"* of adhearing to *"Efficiency Dentistry"* the decay is discovered by the dentist long before it reaches the disease climax in the aching tooth and consequently the fillings are very small and the teeth remain strong and efficient.

Patients who, upon advice of their dentist, faithfully followed this principle of "Efficiency Dentistry" have very few filled teeth and should never have a toothache. They should likewise always be at high efficiency so far as their teeth are concerned. This idea has found favor in industrial institutions where the human health and energy of the operatives has so much to do with the material output. Is it not the logical conclusion that "Efficiency Dentistry" also can work for the maintenance of that human energy which is necessary in maintaining a uniform high grade of material output? Industrial plants employing large numbers of men and women have recognized the idea of health maintenance by installing medical rooms with doctors in charge, recreation and reading rooms with easy chairs and books and periodicals, gymnasiums, roof gardens, billiard rooms, summer outing homes, adequate heating, ventilating and lighting plants, etc., and the operatives have organized base ball teams, card clubs, singing societies and amateur theatricals, also talks and lectures are furnished on salesmanship and shop procedure. Dentistry has been left to the individual, and that, as stated before, is only, except in rare cases, attended to when the ache or pain comes. The dentist eventually gets the patient but what about conserving the energy of the patient in the meantime? The dentist is consulted so late and the decay has gone so far that it means a large filling, extraction of the tooth, or a restoration of some kind as an artificial tooth, crown or bridgework. This calamity is all avoided by "Efficiency Dentistry," and the nerve energy for work is also conserved.

Many large concerns have installed dental offices with dentists on the concerns payroll, whose business it is to care for any dental disabilities that might interrupt the continuity of business operations.

The work in the industrial dental clinic consists of the industrial dentist examining every patient in the plant periodically, indicating on a card index system the dental work needed and checking the work after the patient has had it performed by his or her family dentist. In this way the industrial dental clinic manager discovers the decay and has it attended to by the family dentist or takes care of it himself, and after a few months he has the "Zero System" and "Efficiency Dentistry" well started and bringing results for the good of all concerned.

Is it not possible that more business concerns would adopt this method of conserving the health and of increasing the efficiency of their employees, if the real value of this work as an efficiency agent was better understood? It would be good business for even concerns employing only a few people. In fact, it could very profitably be carried into the service departments of domestic life. It should be a matter of great satisfaction to know that one's servants were free from infectious dental as well as other diseases that might make them a menace, rather than desirably efficient.

The "Zero System," or preventive dentistry, will surely become more a part of the welfare work in manufacturing plants when its benefits to the "human element" are better understood by managers of industrial corporations.

